# 222. Rabies Vaccination to Assess Vaccine Responsiveness after B Cell Targeted Chimeric Antigen Receptor (CAR)-T Cell Therapies: The RAVCAR Study

**DOI:** 10.1093/ofid/ofaf695.080

**Published:** 2026-01-11

**Authors:** Patrick W Flaherty, Lauren Jatt, Elizabeth M Krantz, Anne Konchan, Lalita Priyamvada, Lauren A Greenberg, Karyn A Tindbaek, Molly Briggs, Julian Munoz, Clementine Chalal, Ajay K Gopal, Paul A Carpenter, Mazyar Shadman, Andrew J Cowan, Hans D Ochs, James G Kublin, William Hahn, Subbian Satheshkumar Panayampalli, Michael J Boeckh, Joshua A Hill

**Affiliations:** Fred Hutchinson Cancer Center, Colchester, VT; University of Washington, Seattle, Washington; Fred Hutch Cancer Center, Seattle, Washington; Fred Hutchinson Cancer Center, Colchester, VT; CDC, Atlanta, California; Centers for Disease Control and Prevention, Atlanta, Georgia; Fred Hutchinson Cancer Center, Colchester, VT; Fred Hutchinson Cancer Center, Colchester, VT; Fred Hutchinson Cancer Center, Colchester, VT; Fred Hutchinson Cancer Center, Colchester, VT; Fred Hutchinson Cancer Center, Colchester, VT; Fred Hutchinson Cancer Research Center; University of Washington, Seattle, Washington; Fred Hutchinson Cancer Center, Colchester, VT; Fred Hutchinson Cancer Center, Colchester, VT; Seattle Children's Research Institute, Seattle, Washington; Fred Hutchinson Cancer Center, Colchester, VT; Fred Hutchinson Cancer Center, Colchester, VT; Centers for Disease Control and Prevention, Atlanta, Georgia; Fred Hutchinson Cancer Center, Colchester, VT; Fred Hutch Cancer Center, Seattle, Washington

## Abstract

**Background:**

We conducted an open-label trial using inactivated rabies vaccine (Imovax) as a neoantigen challenge after CAR-T therapy to assess predictors of vaccine response, the utility of a fractional escalating dose prime, and Imovax as a tool to assess ‘vaccine readiness’, even in patients receiving IVIG given that IVIG typically contains no rabies antibodies.

**Figure 1.**
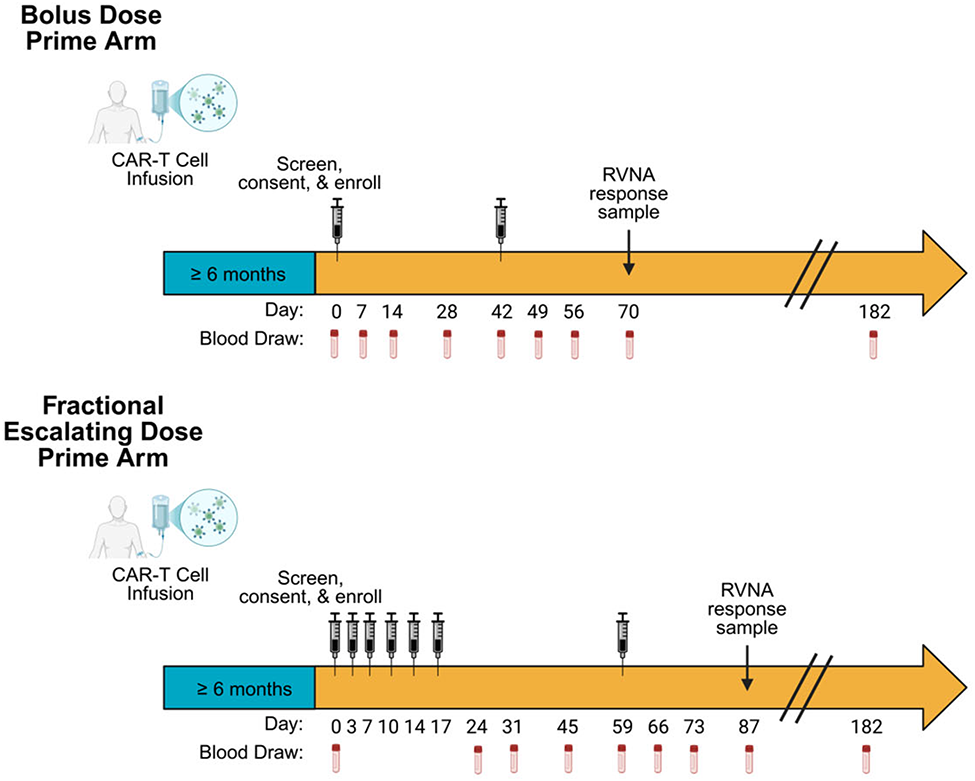
Study design

Blood was collected prior to and at 1, 2, and 4 weeks after completion of primary immunization and boost, and 26 weeks post-primary immunization. Rabies virus neutralizing antibodies (RVNA) were measured at each timepoint. Seroprotective response was defined as RVNA ≥ 0.5 IU/mL at week 4 post-boost, as defined by the World Health Organization (WHO).

**Figure 2.**
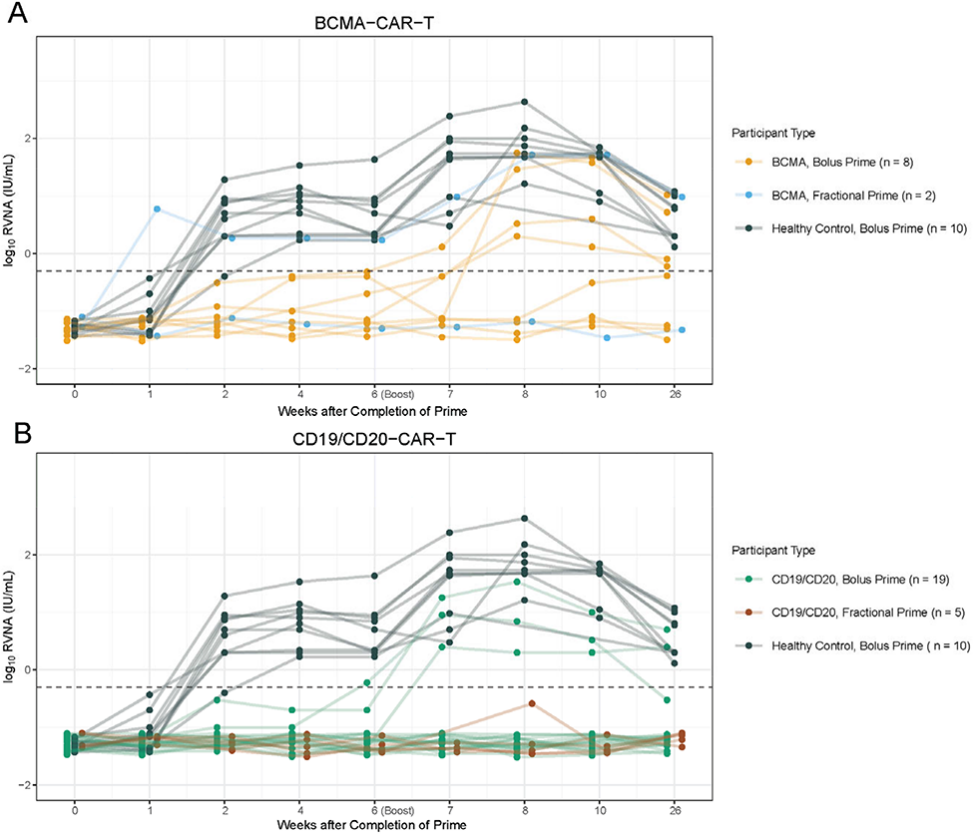
Rabies virus neutralizing antibody (RVNA) kinetics stratified by cohort A-B. RVNA kinetics in 10 healthy volunteers compared to BCMA (panel A) and CD19/CD20-CAR-T recipients (panel B) receiving a bolus prime versus fractional prime. The fractional prime was given as 6 escalating doses over 17 days. The horizontal dashed line indicates the World Health Organization (WHO) threshold for RVNA seroprotection (≥ 0.5 IU/mL).

**Methods:**

We enrolled adults ≥ 6 months post–BCMA or CD19/CD20-CAR-T and healthy controls. Participants received a primary Imovax immunization, as a bolus dose or a fractional series of 6 escalating doses, followed by a bolus boost 6-10 weeks later (Fig 1). Rabies virus neutralizing antibodies (RVNA) were measured before and at 1, 2, and 4 weeks after prime and boost, and 26 weeks post-prime (week 4 post-boost RVNA ≥ 0.5 IU/mL = seroprotective response).

**Figure 3.**
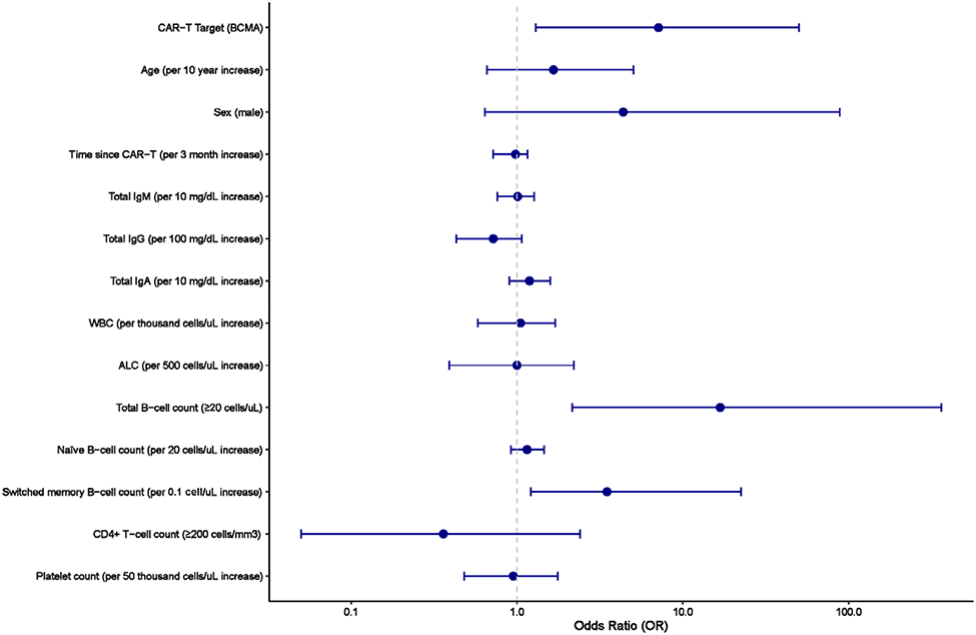
Forest plot of univariate logistic regression model for predictors of rabies virus neutralizing antibody (RVNA) response RVNA response was defined as RVNA ≥ 0.5 IU/mL at 4 weeks post-boost with the inactivated rabies vaccine (Imovax). Odds ratios and confidence intervals > 1 indicate specified predictors are associated with a greater likelihood of RVNA response. Predictor variables were measured at the time of first Imovax dose.

**Figure:**
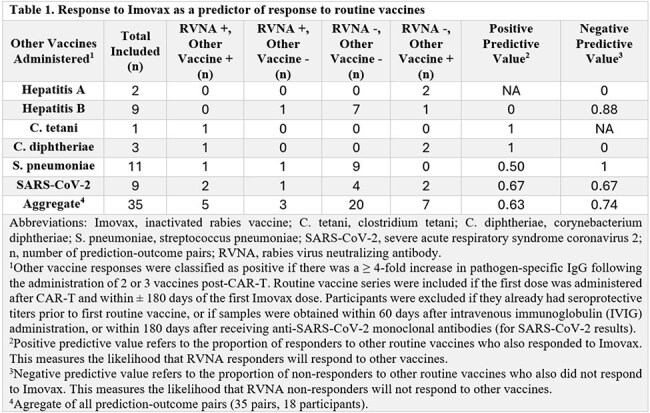

**Results:**

We enrolled 10 controls, 8 BCMA and 19 CD19/CD20-CAR-T recipients in the bolus prime arm, and 2 BCMA and 5 CD19/CD20-CAR-T recipients in the fractional prime arm. The median time from CAR-T to first vaccine was 10 months (IQR, 8-13). Seroprotective responses were observed in all controls, 4/8 (50%) BCMA and 3/19 (16%) CD19/CD20-CAR-T recipients in the bolus prime arm; and in 1/2 (50%) BCMA and 0/5 (0%) CD19/CD20-CAR-T recipients in the fractional prime arm (Fig 2). The RVNA kinetics of the responder in the fractional prime arm appeared similar to healthy controls, with an earlier and higher magnitude response than all other CAR-T recipients. BCMA-CAR-T, B cell count ≥ 20/uL, and higher switched memory B cell count were associated with higher odds of response (Fig 3). RVNA response had a positive predictive value (PPV) of 0.63 and a negative predictive value (NPV) of 0.74 for predicting responses to routine vaccines (Table 1). There were no serious related adverse events, and all participants completed the fractional prime.

**Conclusion:**

We used the rabies vaccine as an *in vivo* immune challenge to interrogate humoral immunocompetence after CAR-T. BCMA-CAR-T recipients were more likely to respond to Imovax than CD19/CD20-CAR-T recipients. A fractional prime could not rescue non-responders but may boost immunogenicity in responders. RVNA response had a moderate PPV and high NPV for predicting responses to routine vaccines and may be a valuable tool for determining vaccine readiness after CAR-T.

**Disclosures:**

Joshua A. Hill, MD, AlloVir: Advisor/Consultant|AlloVir: Grant/Research Support|Century Therapeutics: Advisor/Consultant|CSL Behring: Advisor/Consultant|ExeVir Bio: Advisor/Consultant|GeoVax: Advisor/Consultant|GeoVax: Grant/Research Support|Gilead Australia: Honoraria|Grifols: Advisor/Consultant|Karius: Advisor/Consultant|Karius: Travel|Medscape: Advisor/Consultant|Merck: Grant/Research Support|Moderna, Inc.: Board Member|Modulus: Advisor/Consultant|Oxford immunotec: Grant/Research Support|Sanofi Pasteur Inc.: Advisor/Consultant|Senti BioSciences, Inc: Advisor/Consultant|SymBio: Advisor/Consultant|Takeda: Grant/Research Support|Takeda Netherlands: Honoraria|UpToDate: Royalties

